# Clinical research on liver reserve function by ^13^C-phenylalanine breath test in aged patients with chronic liver diseases

**DOI:** 10.1186/1471-2318-10-23

**Published:** 2010-05-12

**Authors:** Gan-sheng Zhang, Zhi-jun Bao, Jian Zou, Shu-ming Yin, Yi-qin Huang, Hai Huang, De-kai Qiu

**Affiliations:** 1 Department of Gastroenterology, Huadong Hospital, Fudan University, Shanghai 200040, China; 2 Department of Gastroenterology, Renji Hospital, Shanghai Jiaotong University School of Medicine, Shanghai Institute of Digestive Disease, Shanghai 200001, China

## Abstract

**Background:**

The objective of this study was to investigate whether the ^13^C-phenylalanine breath test could be useful for the evaluation of hepatic function in elderly volunteers and patients with chronic hepatitis B and liver cirrhosis.

**Methods:**

L-[1-^13^C] phenylalanine was administered orally at a dose of 100 mg to 55 elderly patients with liver cirrhosis, 30 patients with chronic hepatitis B and 38 elderly healthy subjects. The breath test was performed at 8 different time points (0, 10, 20, 30, 45, 60, 90, 120 min) to obtain the values of Delta over baseline, percentage ^13^CO_2 _exhalation rate and cumulative excretion (Cum). The relationships of the cumulative excretion with the ^13^C-%dose/h and blood biochemical parameters were investigated.

**Results:**

The ^13^C-%dose/h at 20 min and 30 min combined with the cumulative excretion at 60 min and 120 min correlated with hepatic function tests, serum albumin, hemoglobin, platelet and Child-Pugh score. Prothrombin time, total and direct bilirubin were significantly increased, while serum albumin, hemoglobin and platelet, the cumulative excretion at 60 min and 120 min values decreased by degrees of intensity of the disease in Child-Pugh A, B, and C patients (P < 0.01).

**Conclusions:**

The ^13^C-phenylalanine breath test can be used as a non-invasive assay to evaluate hepatic function in elderly patients with liver cirrhosis. The ^13^C-%dose/h at 20 min, at 30 min and cumulative excretion at 60 min may be the key value for determination at a single time-point. ^13^C-phenylalanine breath test is safe and helpful in distinguishing different stages of hepatic dysfunction for elderly cirrhosis patients.

## Background

The liver plays a key role in major metabolic activities and breakdown of many drugs and toxins. The ability to evaluate its functional mass is a very important parameter. Function tests in hepatology should provide accurate information about diagnosis, severity estimation, prognosis assessment and therapy evaluation in patients with liver disease [1]. To improve the conventional static biochemical liver tests (such as ALT, AST, bilirubin, ALP and ALB levels or PT), several dynamic tests have been proposed over recent decades [2,3]. When it comes to the liver metabolic function, the appearance of ^13^CO_2 _in breath after ^13^C-substrate administration means that the administered substance has undergone liver oxidation, thus reflecting the cellular function investigated (microsomal, cytosolic, mitochondrial). ^13^CO_2 _breath test is a simple clinical method for quantifying liver function and is only based on hepatic oxidation of a ^13^C-labeled tracer. As a liver breath test substrate, L-[1-^13^C] phenylalanine is converted to L-[1-^13^C] tyrosine and hidroxylated exclusively in the liver. So L-[1-^13^C] phenylalanine breath test (PheBT) is an easy performed functional liver test that measures cytosolic activity and is independent of hepatic blood flow. Because impaired liver function may limit phenylalanine catabolism, and thereby ^13^CO_2 _production, reduced ^13^CO_2 _release in the exhaled breath is used as an indicator for a disturbed phenylalanine oxidation [4-13]. The basis of PheBT to quantify liver function is that ^13^CO_2 _recovery reflects phenylalanine oxidation within the liver and a reduced oxidation might be used as a marker for hepatocellular dysfunction [14]. Our study also demonstrated that the PheBT can be used as a non-invasive assay to evaluate hepatic function in patients with liver cirrhosis and minimal hepatic encephalopathy [15]. However, age-related reductions in liver weight, volume and blood flow have been reported, as well as a decrease in the number of hepatocytes, changes in hepatocellular structural and functional parameters [16-18]. The purpose of this study is to investigate whether the ^13^C-phenylalanine breath test could be useful for the evaluation of hepatic function in elderly volunteers and patients with chronic hepatitis B and liver cirrhosis.

## Methods

### Patients

All the study subjects underwent a complete history and physical examination, and were > 65 and < 85 years of age. The patients gave their informed consent to participate in the study after a full explanation. Diagnosis of chronic hepatitis B and cirrhosis was made according to the criteria revised in 2005 National Symposium in China [19,20]. The diagnosis of chronic HBV was based on the presence of hepatitis B surface antigen at least 6 months, elevated serum alanine aminotransferase(ALT) levels, HBeAg or anti-HBe positive, with or without HBV DNA as detected by the hybridization method. The diagnosis of cirrhosis was based on clinical findings, laboratory tests, imaging studies, and/or liver histological examination. The cause of liver cirrhosis in patients enrolled in this study was due to Hepatitis B Virus. All patients were classified by the Child-Pugh classification, comprising of ascites, encephalopathy, serum albumin, bilirubin levels and prothrombin time. The Child-Pugh's scores were used to assess the severity of liver cirrhosis.

Exclusion criteria were overt hepatic encephalopathy (HE) or a history of overt HE or neurological or mental diseases; alcohol liver disease or history of recent (<4 weeks) alcohol intake; history of recent (<4 weeks) use of drugs affecting psychometric performances like sedative or other psychotropic drugs and antiviral treatment on chronic hepatitis B; a history of liver transplantation or shunt surgery or transjugular intrahepatic portosystemic shunt for portal hypertension; a history of recent (<4 weeks) gastrointestinal bleeding and electrolyte imbalance; severe medical problems such as congestive heart failure, pulmonary disease, cerebrovascular diseases, diabetes mellitus that could influence breath test. In addition, the patients who took drugs which could possibly alter breath tests results (Corticosteroids, Cimetidine, Diazepam, Omeprazole etc.), patients with thyroid disease, or fever, were excluded; as were patients with any digestive disease which could cause malabsortion.

This study was approved by the hospital ethics committee. Enrollment started September 2006 and ended January 2009, in Huadong Hospital and Renji Hospital. Thirty elderly patients with a diagnosis of chronic hepatitis B and 55 elderly patients with a diagnosis of cirrhosis, confirmed by clinical findings, laboratory tests, imaging studies, and, in this study, 3 patients underwent liver histological examination, were invited to take part in the study. Blood for standard liver tests (serum values of prothrombin time, albumin, total bilirubin, AST, ALT and alkaline phosphatase) was withdrawn immediately before performing the breath test.

### Comparison Groups

A total of 38 elderly healthy volunteers were included as control subjects. Thirty-eight healthy volunteers presented for their yearly physical examinations and had no specific complaints or illness requiring treatment. None of them had a history of liver, respiratory or gastrointestinal malabsortion diseases, neither were they receiving any medication at the time of the study. Biochemical hepatic function, prothrombin time and platelet count were normal in all subjects. Before the study, these subjects were on a normal diet and consumed no drugs for two weeks prior to the test which could affect hepatic enzyme activity.

### Phenylalanine breath test

L-[1-^13^C] phenylalanine (99% in purity, Isotec, USA) was administered orally to the subjects at a dose of 100 mg dissolved in 100 ml of purified water, in the early morning, in the fasting state, 8 h before the PheBT, while the patients were at rest in the sitting position. A 10-ml breath collection tube was used to collect exhaled air. Exhaled air samples were collected in commercially available vacutainer tubes, and the straw method before the drug administration and 10, 20, 30, 45, 60, 90, 120 min after the isotope administration. The value of ^13^CO_2 _in the exhaled air was determined with an air isotope ratio mass spectrometer (Analytical Precision Products, Finnigan, Germany). The ability of the liver to oxidize phenylalanine at each time-point (%^13^C dose h^-1^) was calculated from the level of ^13^CO_2 _in the expired air, on the assumption, based on a report by Schneider et al. that 300 mmol of CO_2 _per m^2 ^of body surface area is generated per unit hour [21]. The cumulative excretion during the 120-min period following the isotope administration was calculated by the integration of the excretion rate curve with time.

### Statistical Analysis

All data were analyzed using SPSS (version 15.0; SPSS, Inc., Chicago, IL). One-way anova by Scheffe's F-test was used for comparison among multiple groups. Correlations with the blood biochemical parameters and parameters of scintigraphy were assessed by determining Pearson's correlation coefficient and Spearman's correlation coefficient. Multiple regression analysis was performed to assess the combined influence of variables on cumulative excretion, ^13^C dose at 20 and 30 min. Intergroup differences were evaluated by the log-rank test. Data were expressed as mean ± SD. *P *value less than 0.05 was considered statistically significant.

## Results

The age range of all the subjects in our study was 66-84 years. All the 30 patients with chronic hepatitis B and 55 patients with liver cirrhosis and the 38 healthy volunteers approached for the study, completed ^13^C-PheBT. The main demographic and clinical data of samples are shown in Table 1. The reason for cirrhosis in patients enrolled in this study was chronic hepatitis B.

**Table 1:** Demographic and Clinical Data of Samples (mean ± SD).

	Control Subjects (n = 38)	Chronic hepatitis B (n = 30)	Cirrhosis (n = 55)
Age	74.1 ± 5.9	74.0 ± 5.3	72.8 ± 5.1
Gender M/F	26/12	21/9	36/19
Height (cm)	164.9 ± 7.8	166.7 ± 7.9	163.5 ± 8.0
Weight (kg)	66.7 ± 9.8	65.3 ± 10.3	62.7 ± 10.2
Child-Pugh Class	-	-	A 19/B 28/C 8

### Comparison of liver function and blood test in every group

Comparison of liver function and blood test in every group is shown in Table 2. Compared with healthy controls, patients with chronic hepatitis B and cirrhosis had worse biochemical parameters, and increasing severity of liver cirrhosis was associated with significantly decreased in serum albumin (ALB), haemoglobin (HB) and platelet count (PLT), and significantly increased in serum total bilirubin (TBIL) and prothrombin time (PT) (P < 0.05).

**Table 2:** Comparison of liver function and blood test in elderly groups (mean ± SD).

Indicatation	Control (n = 38)	Chronic Hepatitis B (n = 30)	Cirrhosis (n = 55)		
			
			Child-Pugh A (n = 19)	Child-Pugh B (n = 28)	Child-Pugh C (n = 8)
ALT (IU/L)	23.3 ± 7.3	90.3 ± 34.5^a^	42.2 ± 17.8 ^a^	52.7 ± 27.8 ^a^	46.3 ± 12.7 ^a^
AST (IU/L)	16.1 ± 5.4	65.4 ± 17.1 ^a^	33.2 ± 11.5 ^a^	43.7 ± 19.8 ^a, c^	51.9 ± 17.1 ^a, b^
GGT (IU/L)	19.6 ± 6.7	23.5 ± 5.8	55.7 ± 33.1 ^a^	59.1 ± 30.1 ^a^	106.0 ± 70.8 ^a, b, d^
ALP (IU/L)	97.3 ± 25.8	101.7 ± 32.2	114.0 ± 58.1	91.8 ± 18.4 ^c^	147.5 ± 63.1 ^a, c, d^
TBIL (μmol/L)	7.9 ± 2.4	9.7 ± 3.4	18.0 ± 5.8 ^a^	22.6 ± 9.4 ^a, c^	64.1 ± 10.5 ^a, b, d^
DBIL (μmol/L)	2.0 ± 0.5	2.3 ± 0.6	7.4 ± 2.7 ^a^	7.3 ± 2.9 ^a^	38.8 ± 2.5 ^a, b, d^
ALB (g/L)	39.9 ± 2.9	39.7 ± 1.9	36.1 ± 2.4 ^a^	30.6 ± 2.1 ^a, b^	25.6 ± 1.1 ^a, b, d^
PT(s)	11.7 ± 1.0	12.1 ± 0.8	14.4 ± 1.5 ^a^	16.7 ± 2.9 ^a, b^	20.9 ± 2.2 ^a, b, d^
HB (g/L)	13.1 ± 1.2	13.2 ± 1.1	11.0 ± 1.1 ^a^	8.5 ± 1.3 ^a, b^	7.9 ± 1.6 ^a, b^
PLT (10^9^/L)	193.3 ± 46.2	175.5 ± 43.2	146.5 ± 41.5 ^a^	91.6 ± 22.5 ^a, b^	90.3 ± 9.9 ^a, b^

Note: ALT, alanine aminotransferase; AST, aspartate aminotransferase; GGT, γ-glutamyltransferase; ALP, alkaline phosphatase; TBIL, serum total bilirubin; DBIL, serum direct bilirubin; ALB, serum albumin; PT, prothrombin time; HB, haemoglobin; PLT, platelet count

Compare to control group, a *P *< 0.01;

Compare with Child-Pugh A: b *P *< 0.01, c *P *< 0.05;

Compare between Child-Pugh B and C, d *P *< 0.05

### Comparison of %^13^C dose h^-1 ^curves and %^13^C cumulative excretion curves after oral administration of L-[1-^13^C] Phenylalanine in every group

**Figure 1:**
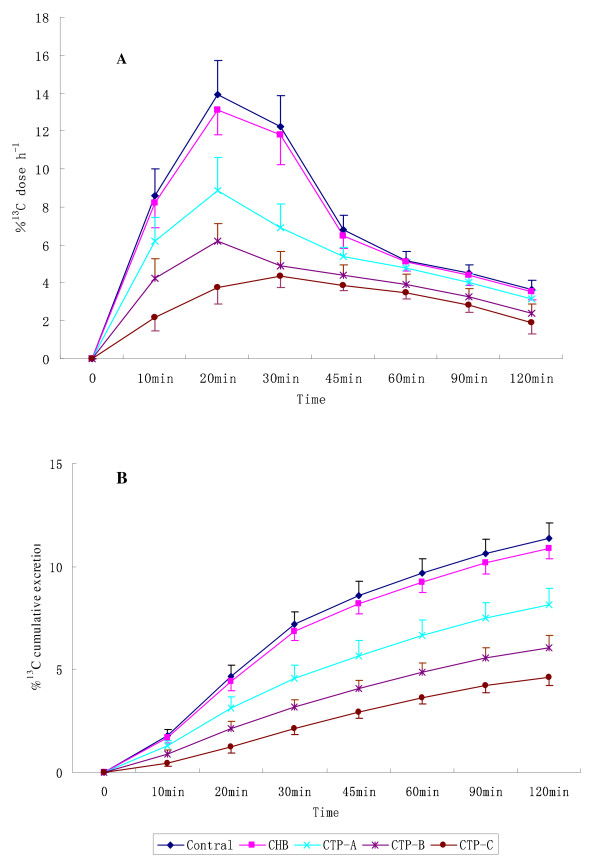
**%^13^C dose h^-1 ^curves (A) and %^13^C cumulative excretion curves (B) after oral administration of L-[1-^13^C] Phenylalanine in elderly groups**. Note: CHB, chronic hepatitis B; CTP, Child-Pugh;

The %^13^C dose h^-1 ^at 20 min and %^13^C dose h^-1 ^at 30 min combined with the cumulative excretion at 60 min and 120 min showed correlations with the chronic liver diseases, especially Child-Pugh score (Table 3, Figure 1A and 1B). The cumulative excretion at 60 min and 120 min values decreased by degrees of intensity of the disease in healthy controls and Child-Pugh A, B, and C patients (*P *< 0.01).

**Table 3:** Comparison of %^13^C dose h^-^^1 ^curves and %^13^C cumulative excretion curves after oral administration of L-[1-^13^C] Phenylalanine in elderly groups (mean ± SD).

Time	Control (n = 38)	Chronic Hepatitis B (n = 30)	Cirrhosis (n = 55)		
			
			Child-Pugh A (n = 19)	Child-Pugh B (n = 28)	Child-Pugh C (n = 8)
^13^CO_2_ER _t_					
10 min	8.6 ± 1.4	8.2 ± 1.3	6.2 ± 1.3 ^a^	4.3 ± 1.0 ^a, c^	2.2 ± 0.7 ^a, c, d^
20 min	13.9 ± 1.8	13.1 ± 1.3	8.9 ± 1.7 ^a^	6.2 ± 0.9 ^a, c^	3.7 ± 0.8 ^a, c, d^
30 min	12.3 ± 1.6	11.8 ± 1.6	6.9 ± 1.2 ^a^	5.1 ± 0.6 ^a, c^	4.1 ± 0.3 ^a, c, e^
45 min	6.8 ± 0.8	6.5 ± 0.7	5.4 ± 0.5 ^a^	4.4 ± 0.5 ^a, c^	3.9 ± 0.3 ^a, c, e^
60 min	5.2 ± 0.5	5.1 ± 0.5	4.8 ± 0.5 ^a^	3.9 ± 0.5 ^a, c^	3.5 ± 0.3 ^a, c, e^
90 min	4.5 ± 0.5	4.4 ± 0.5	4.0 ± 0.4 ^a^	3.2 ± 0.5 ^a, c^	2.8 ± 0.4 ^a, c, e^
120 min	3.7 ± 0.5	3.6 ± 0.5	3.1 ± 0.4 ^a^	2.4 ± 0.5 ^a, c^	1.9 ± 0.6 ^a, c, e^
^13^C_CUM t_					
CUM 10 min%	1.8 ± 0.3	1.7 ± 0.3	1.3 ± 0.3 ^a^	0.9 ± 0.2 ^a, c^	0.5 ± 0.1 ^a, c, d^
CUM 20 min%	4.7 ± 0.5	4.4 ± 0.4	3.1 ± 0.6 ^a^	2.2 ± 0.3 ^a, c^	1.2 ± 0.3 ^a, c, d^
CUM 30 min%	7.2 ± 0.6	6.9 ± 0.4	4.5 ± 0.7 ^a^	3.2 ± 0.3 ^a, c^	2.1 ± 0.3 ^a, c, d^
CUM 45 min%	8.6 ± 0.7	8.2 ± 0.5	5.7 ± 0.7 ^a^	4.1 ± 0.4 ^a, c^	2.9 ± 0.3 ^a, c, d^
CUM 60 min%	9.7 ± 0.7	9.3 ± 0.5	6.6 ± 0.8 ^a^	4.9 ± 0.5 ^a, c^	3.6 ± 0.3 ^a, c, d^
CUM 90 min%	10.6 ± 0.7	10.2 ± 0.5	7.5 ± 0.8 ^a^	5.6 ± 0.5 ^a, c^	4.2 ± 0.4 ^a, c, d^
CUM 120 min%	11.4 ± 0.8	10.9 ± 0.5	8.1 ± 0.8 ^a^	6.1 ± 0.6 ^a, c^	4.6 ± 0.4 ^a, c, d^

Note: Compare to control group, a *P *< 0.01, b *P *< 0.05;

Compare with Child-Pugh A: c *P *< 0.01;

Compare between Child-Pugh B and C, d *P *< 0.01, e *P *< 0.05

More ^13^CO_2 _was exhaled by healthy subjects than by those with disease and, in healthy controls, ^13^CO_2 _peaked at 20-30 min after the administration of the study drugs. Cumulative measurements were also investigated and offered no benefit over single measurements at 60 min. Cumulative excretion should combine with the %^13^C dose h^-1 ^at 20 min and %^13^C dose h^-1 ^at 30 min for application.

### Coefficients of Correlation between PheBT Results and Blood Biochemical Parameters

Table 4 shows the correlation between the %^13^C dose h^-1 ^at 20, 30 min and %^13^C cumulative excretion at 60, 120 min (the most important parameters) and biochemical liver function tests. ALB, HB, TBIL, DBIL, PT, PLT and Child-Pugh score were highly correlated with the ^13^CO_2_ER _20_, ^13^CO_2_ER _30 _and ^13^C_CUM 60_.

**Table 4:** Coefficients of Correlation between PBT Results and Blood Biochemical Parameters.

	^13^CO_2_ER _20_		^13^CO_2_ER _30_		^13^C_CUM 60_		^13^C_CUM 120_	
				
	R	*P *velue	r	*P *velue	r	*P *velue	r	*P *velue
ALT	0.01	0.87	0.01	0.89	-0.00	0.99	-0.00	0.96
AST	-0.17	0.07	-0.13	0.15	-0.17	0.05	-0.18	0.04
GGT	-0.61	<0.01	-0.59	<0.01	-0.63	<0.01	-0.63	<0.01
ALP	-0.17	0.07	-0.09	0.32	-0.15	0.09	-0.16	0.08
TBIL	-0.71	<0.01	-0.63	<0.01	-0.70	<0.01	-0.71	<0.01
DBIL	-0.65	<0.01	-0.58	<0.01	-0.65	<0.01	-0.66	<0.01
ALB	0.80	<0.01	0.81	<0.01	0.84	<0.01	0.85	<0.01
PT	-0.80	<0.01	-0.74	<0.01	-0.80	<0.01	-0.81	<0.01
HB	0.80	<0.01	0.83	<0.01	0.85	<0.01	0.85	<0.01
PLT	0.72	<0.01	0.61	<0.01	-0.71	<0.01	-0.70	<0.01
Child-Pugh score	-0.81	<0.01	-0.67	<0.01	-0.83	<0.01	-0.84	<0.01

Note: ALT, alanine aminotransferase; AST, aspartate aminotransferase; GGT, γ-glutamyltransferase; ALP, alkaline phosphatase; TBIL, serum total bilirubin; DBIL, serum direct bilirubin; ALB, serum albumin; PT, prothrombin time; HB, haemoglobin; PLT, platelet count

### The safety of ^13^C-PheBT

No side effects such as abdominal pain, abdominal swelling and other signs were observed in any volunteer or patient, hepatic and renal functions did not significantly change after oral intake of L-[1-^13^C] phenylalanine.

## Discussion

Quantitative evaluation of hepatocellular function is important from the aspect of mortality prediction following surgery for liver cirrhosis and from that of the prognosis in cases of acute hepatitis. Although several methods have been used to quantitatively evaluate the reserve capacity of the liver, e.g. methods evaluating clearance of galactose and indocianine, ascialoglycoprotein receptor of the liver, and breath tests, no definitive method has been established as yet. Some possible reasons for the difficulty of establishing a definitive method for the evaluation of liver function are absence of any entirely safe substrates, the complexity of the tests, and the invasiveness of the methods. In this regard, breath tests are safe and non-invasive as a stable isotope is used as a tracer and simple collection of expired air samples allows the determination.

Phenylalanine is one of the eight essential amino acids for humans and must be obtained from dietary sources. This amino acid is mainly decomposed in the liver, but with a low extraction from plasma into the liver (E = 0.2), that is even lower in patients with liver diseases [22-25]. Therefore, as a breath test substrate, phenylalanine provides a good index of hepatic metabolic capacity. Furthermore, phenylalanine has the additional benefits that its metabolism is largely unaffected by blood flow rearrangement and it has neither pharmacological nor allergic properties. These properties make ^13^C-phenylalanine an ideal breath test substrate to assess liver function [1]. Phenylalanine oxidation, including hydroxylation to tyrosine and further degradation, occurs in the hepatic cytosol. During degradation, the position 1 carbon of phenylalanine is decarboxylated, with the production of CO_2_, which is released in the breath. In the case of L-[1-^13^C] phenylalanine being used, ^13^CO_2 _is detected as the final metabolite in the breath.

Burke et al [4] was the first to utilize PheBT to determine the hepatocyte functional capacity in patients with end-stage liver diseases. They found that ^13^CO_2 _excretion decreased by up to 80% after intake of L-[1-^13^C] phenylalanine. In recent years, similar results were subsequently obtained in different populations [5-13], including our study [15], however, seldom in elderly populations [26]. Additional estimation of phenylalanine hydroxylase activity by the ^13^C-PheBT might be useful in that it evaluates the real-time condition in patients.

Results emerging from the present study show that PheBT could discriminate liver function not only between healthy subjects and liver disease patients, but also between different stages of cirrhosis patients. Based on the reference [4-13,27], we selected 8 different time point to collect exhaled air. The %^13^C dose h^-1 ^curves showed the peaks were obvious in group normal and chronic hepatitis B, and the peak appeared at 10-30 min after phenylalanine administration, whereas, the curves decreased along with the severity of liver disease in group cirrhosis. The %^13^C dose h^-1 ^at 20 min and 30 min combined with the cumulative excretion at 60 min and 120 min showed correlations with serum albumin, hemoglobin, platelet, prothrombin time, total and direct bilirubin and Child-Pugh score. With comprehensive analysis of the results, the exhaled air samples might be only collected at 5 different time point (before the drug administration and 10, 20, 30, 60 min after the isotope administration) as same as the adults. As the primary indication, the %^13^C dose h^-1 ^at 20 min and 30 min combined with the cumulative excretion at 60 min and 120 min not only could cut the breath test time, but were correlated with serum TBIL, ALB, PT and Child-Pugh score. It is shown that ^13^C-PheBT could reflect compensation of liver function, especially reflect liver compensatory function quantitatively. The results were a beneficial complementary for liver function evaluation. Therefore, PheBT is a safe, sensitive test and easy to prepare, perform and analyses, especially in elderly.

Nowadays, the Child-Pugh classification remains the most widely accepted tool as a measure of disease severity in liver disease. However, in addition to being safe, non-invasive, easy to perform, and well correlated with the Child-Pugh score, PheBT could provide additional information in that direct hepatocyte functional capacity is measured, avoiding any subjectivity in hepatic dysfunction staging and also avoiding other elements that could modify the real degree of hepatic damage. The function tests can provide different information from that obtained by a liver biopsy, but not such detailed information on hepatic disease as that provided by histological examination. Furthermore, function tests cannot indicate the extent of inflammation or the degree of fibrosis.

Several factors have prevented widespread adoption of the 13C-PheBT: it is more expensive and time and labor consuming than the conventional biochemical tests. But, it is clear that such tests are superior to the tranditional Child-Pugh score in predicting survival or life-threatening complication [28,29]. PheBT is able to discriminate hepatic functional capacity not only between healthy individuals and patients with liver disease, but also between different stages of hepatic dysfunction in adult and elderly. Our results show that 13C-PheBT can distinguish between class A cirrhotics and those with more advanced disease, but does not appear to be able to distinguish sufficiently between class B and C cirrhotics. Well-controlled, prospective trials are needed to clearly define the role of these quantitative tests in the management of patients with liver disease.

## Conclusions

In summary, the conclusion of our study is that ^13^C-PheBT can be used as a non-invasive assay to evaluate hepatic function in elderly patients with liver cirrhosis. The %^13^C dose h^-1 ^at 20 min, %^13^C dose h^-1 ^at 30 min and cumulative excretion at 60 min might be the key value for determination at a single time-point. The ^13^C-PheBT is safe and helpful in distinguishing different stages of hepatic dysfunction for elderly cirrhosis patients.

## Competing interests

The authors declare that they have no competing interests.

## Authors' contributions

GSZ, ZJB, SMY, JZ and DKQ participated in the design of the study; GSZ, ZJB, SMY, YQH and HH carried out the research; GSZ, SMY, YQH and HH performed the statistical analysis; and ZJB and JZ wrote the paper. All authors read and approved the final manuscript.

## Pre-publication history

The pre-publication history for this paper can be accessed here:

http://www.biomedcentral.com/1471-2318/10/23/prepub
